# *PRNP* expression predicts imaging findings in sporadic Creutzfeldt‐Jakob disease

**DOI:** 10.1002/acn3.51739

**Published:** 2023-02-06

**Authors:** Iris J. Broce, Eduardo Caverzasi, Simone Sacco, Ryan Michael Nillo, Matteo Paoletti, Rahul S. Desikan, Michael Geschwind, Leo P. Sugrue

**Affiliations:** ^1^ Weill Institute for Neurosciences, Department of Neurology University of California, San Francisco, UCSF San Francisco California USA; ^2^ Department of Neurosciences University of California, San Diego San Diego California USA; ^3^ Department of Brain and Behavioral Sciences University of Pavia Pavia Italy; ^4^ Division of Neuroimaging, Department of Medical Imaging University of Toronto Toronto Ontario Canada; ^5^ Neuroradiology Section, Department of Radiology and Biomedical Imaging University of California, San Francisco San Francisco California USA; ^6^ Advanced Imaging and Radiomics Center, Neuroradiology Department IRCCS Mondino Foundation Pavia Italy

## Abstract

**Objective:**

We explored the relationship between regional *PRNP* expression from healthy brain tissue and patterns of increased and decreased diffusion and regional brain atrophy in patients with sporadic Creutzfeldt‐Jakob disease (sCJD).

**Methods:**

We used *PRNP* microarray data from 6 healthy adult brains from Allen Brain Institute and T1‐weighted and diffusion‐weighted MRIs from 34 patients diagnosed with sCJD and 30 age‐ and sex‐matched healthy controls to construct partial correlation matrices across brain regions for specific measures of interest: *PRNP* expression, mean diffusivity, volume, cortical thickness, and local gyrification index, a measure of cortical folding.

**Results:**

Regional patterns of *PRNP* expression in the healthy brain correlated with regional patterns of diffusion signal abnormalities and atrophy in sCJD. Among different measures of cortical morphology, regional patterns of local gyrification index in sCJD most strongly correlated with regional patterns of *PRNP* expression. At the vertex‐wise level, different molecular subtypes of sCJD showed distinct regional correlations in local gyrification index across the cortex. Local gyrification index correlation patterns most closely matched patterns of *PRNP* expression in sCJD subtypes known to have greatest pathologic involvement of the cerebral cortex.

**Interpretation:**

These results suggest that the specific genetic and molecular environment in which the prion protein is expressed confer variable vulnerability to misfolding across different brain regions that is reflected in patterns of imaging findings in sCJD. Further work in larger samples will be needed to determine whether these regional imaging patterns can serve as reliable markers of distinct disease subtypes to improve diagnosis and treatment targeting.

## Introduction

Creutzfeldt‐Jakob disease (CJD) is a rapidly progressive, fatal neurodegenerative disorder caused by the accumulation of abnormally folded “prion‐related protein” (PrP) within the brain leading to neuronal loss, vacuolation, and gliosis. The term prion—for “proteinaceous infectious particle”—emphasizes that the misfolded protein is sufficient to cause or transmit the disease.^1^, ^2^ In practice, however, CJD is rarely acquired through transmission (<1%), with most cases being either genetic (5%–15%) or sporadic CJD (sCJD; 85%–90%) in origin.^2^, ^3^ Most forms of genetic prion disease occur in the 50s to 60s and are caused by germline mutations in the prion protein gene, *PRNP*, which predispose the encoded normal “cellular” form of the prion protein (PrP^C^) to misfold into the pathologic “scrapie” form (PrP^Sc^).^4^ The peak age of onset for sCJD is later, in the mid 60s,^2^, ^3^ and patients with sCJD generally die within a year of diagnosis, with a mean survival of 4–8 months.^3^, ^5^, ^6^ In sCJD, somatic mutations in *PRNP* or environmental factors are presumed to trigger PrP^C^ misfolding. Patients with sCJD can be classified into six molecular subtypes depending on the methionine/valine (M/V) genotype at codon 129 of the *PRNP* allele and the molecular weight of the proteinase‐resistant PrP^Sc^ fragments—21 kDA for type 1 and 19 kDA for type 2.^3^ Most patients with sCJD have either type 1 or type 2 fragments (although a minority have a combination of both types, providing three additional molecular subtypes: MM1/2, MV1/2, VV1/2).^3^

Definite diagnosis of sCJD requires pathologic analysis of brain tissue to detect the misfolded protein and characteristic pathological changes.^3^, ^7^, ^8^ In current practice, however, MRI—particularly, diffusion imaging—is a key part of sCJD diagnosis.^9^, ^10^ Early in the disease course, high signal intensity on diffusion‐weighted imaging (DWI) (i.e., reduced mean diffusivity) within the cortex and deep gray nuclei provides a relatively sensitive and specific imaging marker for CJD.^9^, ^10^, ^11^, ^12^, ^13^ As the disease progresses, however, mean diffusivity generally increases within affected areas, becoming “pseudo‐normal” or elevated, likely reflecting the ongoing gliosis and neuronal loss that ultimately manifest as atrophy in later stage disease.^14^, ^15^ Within individual patients, imaging findings may be spatially heterogeneous across the brain, with different regions exhibiting findings that suggest earlier or later stages of disease. Despite this heterogeneity, certain clinical and molecular subtypes of CJD have been associated with characteristic imaging findings,^9^, ^16^ suggesting that imaging patterns might provide endophenotypes for distinct disease subtypes. Nevertheless, our understanding of why certain brain regions are selectively vulnerable to involvement in sCJD^16^, ^17^ and how that vulnerability relates to different molecular subtypes and clinical disease phenotypes is lacking.^5^, ^6^ Understanding the genetic environment in which prion protein misfolding occurs and how it relates to regional disease vulnerability and associated imaging findings has the potential to provide insights into sCJD pathophysiology and to inform new diagnostic and prognostic tools. As a step toward developing these imaging‐genetics connections, here we explore the relationship between patterns of regional brain *PRNP* expression and imaging findings in sCJD.

## Methods

### *PRNP* expression in the adult human brain

We investigated regional patterns of *PRNP* expression in the healthy brain across cortical and subcortical areas using *PRNP* microarray gene expression data from the Allen Brain Sciences Institute (ABI; www.brain‐map.org), a publicly available microarray dataset widely used for exploration of gene networks in the human brain. The microarray data were sampled from six adult human donors (3 Caucasian, 2 African American, 1 Hispanic, aged 24–57 years) in approximately 400–500 tissue samples from each donor, either in the left hemisphere only (*n* = 4) or in both hemispheres (*n* = 2). Although the same anatomical regions were sampled in all six donors, both the exact number and position of samples varied among donors. Donors also differed in other characteristics, including cause of death, post‐mortem intervals, brain PH, tissue cytoarchitectural integrity, RNA quality, and number of probes used for each gene. Given space constraints, we refer the reader to the original technical white paper for additional details regarding the dissection methods, quality control, and normalization measures (https://www.google.com/url?sa=t&rct=j&q=&esrc=s&source=web&cd=&ved=2ahUKEwiT7NfSlfP8AhVmOUQIHSlOB-oQFnoECA8QAQ&url=http%3A%2F%2Fhelp.brain-map.org%2Fdownload%2Fattachments%2F2818165%2FWholeBrainMicroarray_WhitePaper.pdf&usg=AOvVaw1vQMgepnNTA7t9IGUHiYlM).

The Allen Brain Institute provides normalized data as well as raw, log2 transformed data. The expression data were normalized within donors to account for array related and dissection related biases and differences in RNA integrity number and across donors to allow for comparison across brains (http://help.brain‐map.org/download/attachments/2818165/Normalization_WhitePaper.pdf).^18^, ^19^ We downloaded expression values (normalized within and across donors) and averaged the expression values for each region over the two available *PRNP* probes: A_23_P109143 and A_24_P914134. Exome data, including *PRNP* codon 129 polymorphisms, unfortunately, were not available.

### sCJD cases and healthy control samples

**Figure 1:**
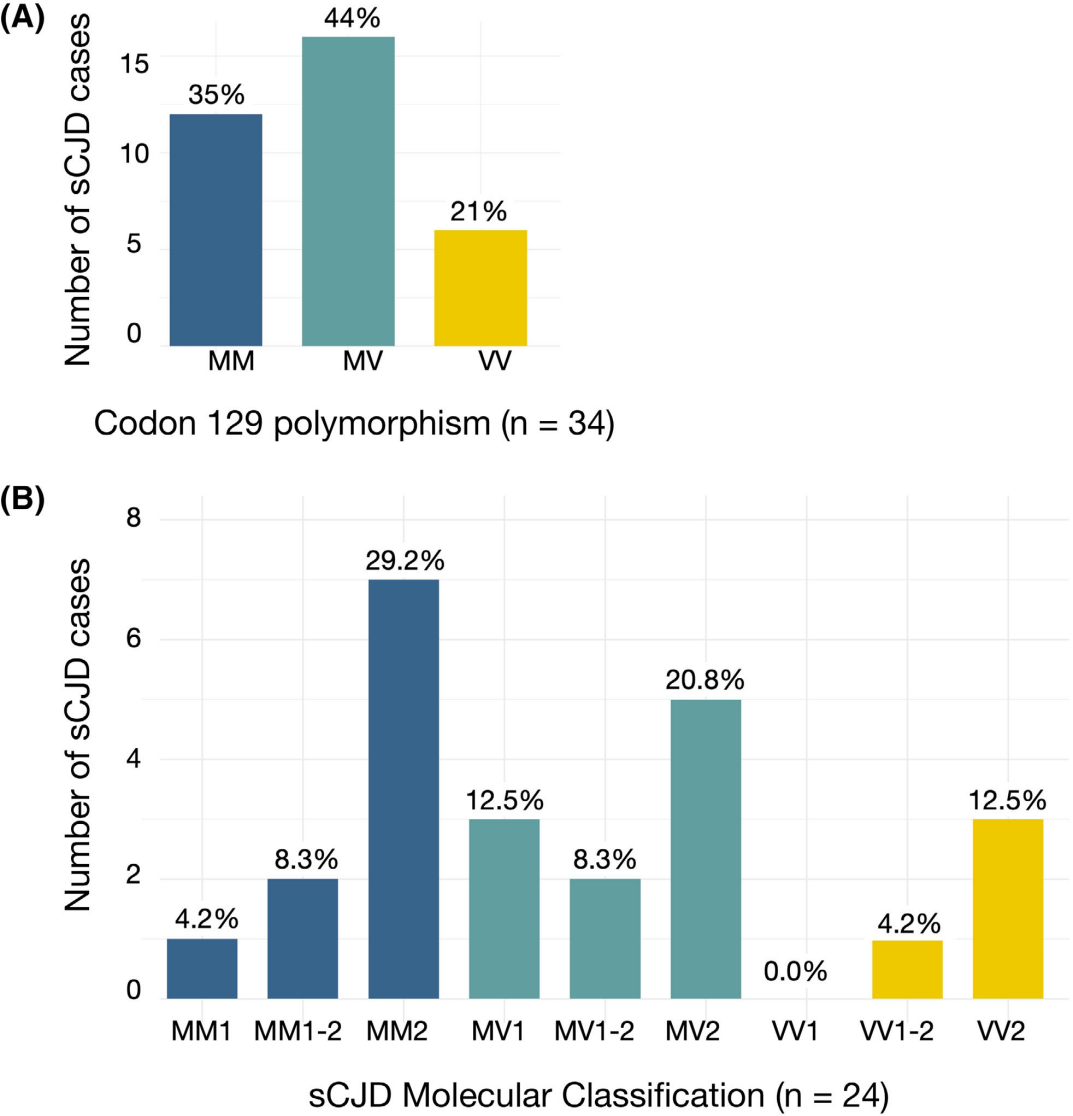
Histograms showing distribution of *PRNP* codon 129 polymorphism and sCJD molecular classification. (A) Methionine/valine (M/V) genotype information at codon 129 for all 34 sCJD cases (B) M/V genotype at codon 129 and molecular classification for the 24 pathology‐proven sCJD cases.

MRIs and clinical data were available for 37 sCJD participants.^14^ Three participants were excluded from further analysis because their T1‐weighted imaging showed excessive motion artifact or atrophy for the planned analyses. Thus, we evaluated 3D‐ volumetric T1‐weighted and high angular resolution diffusion‐weighted imaging (HARDI) from 34 patients diagnosed with sCJD and 30 age and sex matched healthy controls from the Memory and Aging Center at the University of California, San Francisco (UCSF MAC) (Table 1). The participants with sCJD were evaluated in the UCSF MAC clinical research program from 06/2008 to 09/2015, with 71% (*n* = 24) ultimately pathologically proven by brain autopsy, and the remaining 29% (*n* = 10) meeting UCSF probable sCJD criteria.^13^, ^20^ The distribution of sCJD cases by molecular classification is shown in Fig. 1. Of note, all MM2 cases were clinically MM2‐cortical (MM2‐C) subtypes (i.e., not thalamic) based on their diffusion MRI and clinical presentations. All MRI scans were acquired on a 3T Siemens Tim Trio scanner. For participants with multiple scans, the earliest scan with the best quality was selected. Detailed information on participant inclusion criteria and imaging parameters can be found in prior reports.^17^, ^21^, ^22^ The UCSF Committee on Human Research approved the procedures for all participants. All participants or their surrogates provided informed consent prior to participation.

**Table 1:** Clinical features of sporadic Creutzfeldt‐Jakob patients and controls.

	Controls *n* = 30	sCJD *n* = 34
Age at first evaluation, years, mean ± SD (median, range)	64 ± 10 (66, 45–78)	64.88 ± 7.21 (65.5, 49–82)
Sex, female (%)	50	44
Right‐handed (%)	100	97
Time‐Ratio mean ± SD (median, range)		0.61 ± 0.24 (0.67, 0.11–1)
Total disease duration, months, mean ± SD (median, range)		16.30 ± 8.14 (17, 3–34)
MMSE score, mean ± SD (median, range)	29.4 ± 1 (30, 26–30)	15.12 ± 9.35 (18, 0–27)
Barthel index, mean ± SD (median, range)		73.8 ± 30 (80, 0–100) (*n* = 30)
CDR‐SOB, mean ± SD (median range)		10 ± 5.68 (1–18) (*n* = 25)
Pathologically confirmed cases (*n*; %)		27; 79%

Time‐Ratio: time from onset of symptoms to MRI scan/total disease duration.

CDR‐SOB, clinical dementia rating scale sum of boxes scores; MMSE, mini‐mental state examination; SD, standard deviation.

### MRI processing

The HARDI dataset was acquired using a single‐shot spin‐echo echo‐planar imaging (EPI) sequence including 55 contiguous axial slices acquired in an interleaved order with the following parameters: TR/TE = 8000/109 ms; flip angle = 90°; matrix = 100 × 100; in‐plane resolution = 2.2 mm^2^; slice thickness = 2.2 mm; 64 non‐collinear diffusion sensitization directions at *b* = 2000 s/mm^2^, 1 at *b* = 0; integrated parallel acquisition technique acceleration (IPAT) factor = 2. A high resolution T1‐weighted image (TR/TE = 2300/2.98 ms; flip angle = 9°; slice voxel = 1 mm^3^; FoV = 256 mm) was acquired for all participants for structural reference, for purpose of normalization, and for deriving morphological measures.

Cortical and subcortical reconstruction and volumetric segmentation of all T1‐weighted MRI scans was performed using the FreeSurfer software package, version 6.0 (http://surfer.nmr.mgh.harvard.edu/).^23^ Regions of interest were defined by the Desikan–Killiany atlas.^24^ We quantified disease burden using morphological measures of volume, cortical thickness, and local gyrification index (*l*GI). *l*GI is an advanced metric that measures the amount of cortex buried in sulcal folds and is defined as the ratio between the cortical area buried within sulcal folds and that on the gyral surface.^25^ A cortex with extensive folding has a large gyrification index, whereas a cortex with limited folding has a small gyrification index. For all diffusion‐weighted scans, we computed mean diffusivity (MD) as the average across all 64 non‐collinear diffusion sensitization directions, and registered these MD maps to standard space (MNI 152).^14^ All measures were corrected for head size. We then used FreeSurfer to register these standardized MD maps to FreeSurfer's 3D surfaces allowing for both ROI‐based analysis using the Desikan–Killiany atlas as well as whole brain analysis at the vertex level. To compare regional expression data from Allen Brain Science Institute (ABI) to imaging data, which were parcellated using the Desikan–Killiany atlas in Freesurfer,^24^ we mapped each individual anatomical label provided by ABI to the corresponding anatomical label for that location in Freesurfer. Two neuroradiologists (LS and RD) performed the mapping between ABI and Freesurfer regions.

ABI and Freesurfer each provide coarse and fine level anatomical labels. For each level of granularity, we restricted our analyses to regions shared across *PRNP* expression and imaging measures. Regions with Freesurfer anatomic labels that lacked a corresponding anatomical label within the ABI data were excluded from all analysis; these included: superior temporal sulcus, pericalcarine cortex, and entorhinal cortex. Regions with missing ABI *PRNP* expression data from one or more donors were also excluded, these included: caudal middle frontal gyrus, rostral middle frontal gyrus, frontal pole, temporal pole, caudal anterior cingulate, and posterior cingulate. At the coarse‐grained level we correlated regional *PRNP* expression with regional volume and MD, therefore, both cortical and subcortical regions were included in this analysis. At the fine‐grained level we examined correlations between regional *PRNP* expression and regional volume, MD, cortical thickness, and local gyrification index (*l*GI). *lGI* is a cortical measure and Freesurfer does not compute *l*GI values for deep gray nuclei (caudate, putamen, pallidum, thalamus) or the hippocampus, therefore, these regions were excluded from our fine‐grained, *l*GI, analyses. The final sample included diffusion data, and volumetric, cortical thickness, and *l*GI data from 34 sCJD cases and 30 controls.

### Statistical analysis

We explored the association between regional patterns of *PRNP* expression in healthy brains and regional patterns of sCJD associated imaging findings, including morphological and diffusion signal abnormalities, in sCJD cases and healthy controls. We performed partial correlation network analysis separately for cases and healthy controls to assess for regional spatial correlations in each measure of interest (i.e., *PRNP* expression, volume, cortical thickness, *l*GI, and mean diffusivity), for example, to assess if *PRNP* expression in one region across a cohort correlated with *PRNP* expression in other region(s). To evaluate similarity between regional correlations in *PRNP* expression and imaging measures we used the Mantel test^26^: a permutation test of the correlation between two matrices that randomly permutes the rows and columns of one matrix to generate a *z*‐statistic for the inter‐matrix correlation under the null hypothesis that the matrices are unrelated. In all analyses, we randomly permuted the imaging correlation matrix 10,000 times. R 3.3.2 statistical software was used for all analyses (http://www.R‐project.org).

As each measure of interest has different ranges and distributions, to capture relative patterns of expression, morphology, and diffusion, all expression and imaging measures were *z*‐scored with respect to the mean and standard deviation of controls within each region and across participants before computing the correlations. For each partial correlation analysis, we computed the Pearson correlation coefficient between the *z*‐scores for each pair of regions for the measure of interest, controlling for correlation with the average of the *z*‐scores across all other regions. Because imaging findings in sCJD are often asymmetric,^16^, ^17^ expression and imaging measures from the two hemispheres were treated as independent samples.

### Surface‐based (vertex‐wise) correlation analyses across the cerebral cortex

Our analysis of regional variation in *PRNP* expression in healthy controls and imaging measures in sCJD cases compared to controls revealed patterns of correlation in imaging measures across the brain in sCJD cases that mirrored those in *PRNP* expression (see Results). In the cerebral cortex (where the highest resolution expression data were available) similarities in regional correlation patterns between *PRNP* expression and sCJD imaging were strongest for the *l*GI measure. To further explore these patterns of anatomic correlation across the cerebral cortex, we performed a post‐hoc vertex‐wise cortical analysis, mapping anatomic correlations in *l*GI in sCJD cases and healthy controls across the 163,842 Freesurfer vertices in each hemisphere.^27^ Specifically, for cases and controls, we evaluated the correlation between the mean *l*GI within a “seed” region and *l*GI at every other vertex across the cerebral cortex.^28^ In this case, the “seed” regions were defined by clusters of regions that showed strong intra‐cluster positive correlation and strong inter‐cluster negative correlation in the *PRNP* cortical correlation matrix. As with the methodology used in our regional partial correlation analyses, in this analysis, *l*GI values from the two hemispheres were treated independently and then results were averaged by registering the right hemisphere of each subject to the left hemisphere using FreeSurfer's interhemispheric registration method.^29^

Given prior work showing that certain molecular subtypes of sCJD show different regional patterns of change on DWI, such as MV2 and VV2 primarily affecting deep gray nuclei, MM2‐C primarily affecting the cortex, and MM1 and MV1 affecting both (we had no known VV1 cases),^16^, ^30^ we further assessed whether vertex‐wise anatomic correlations in *l*GI across the cerebral cortex differed between molecular subtype groups. To facilitate visual comparison of the resulting average correlation maps between groups, we normalized the vertex‐wise coefficients by the top decile of absolute correlations within each group.

## Results

### *PRNP* expression is highest in the neocortex and lowest in the hippocampus

**Figure 2:**
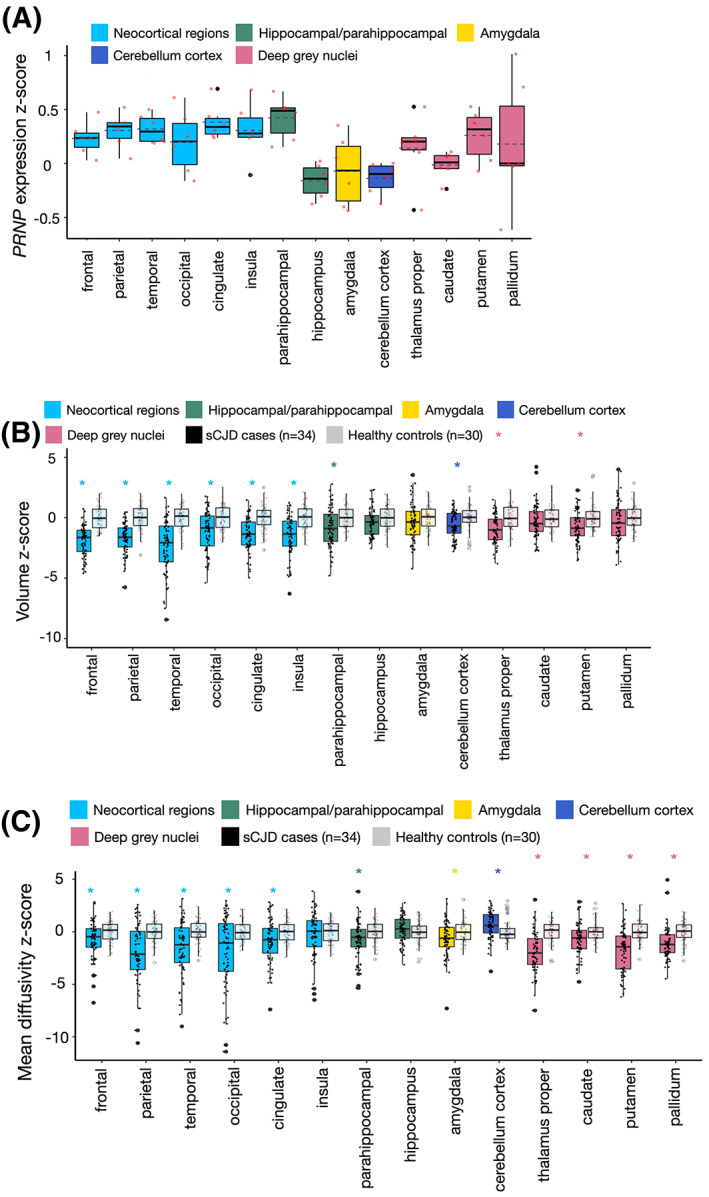
Jitter boxplots of cortical and subcortical regional *PRNP* expression and imaging measures. (A) *PRNP* expression in healthy individuals (*z*‐scored across all regions, participants, and *PRNP* probes). (B) Volumes in patients with sCJD and healthy controls. (C) Mean diffusivity in patients with sCJD and healthy controls. Imaging measures are *z*‐scored within each region using the mean and standard deviation of healthy controls. *p ≤ .05. The *x*‐axis is labeled with respect to brain structure.

Figure 2A shows how *PRNP* expression varies across cortical and subcortical regions and the cerebellar cortex. There was significantly elevated average expression within all neocortical regions (i.e., frontal, temporal, parietal, occipital, insula, cingulate) compared to hippocampus (*p* = 0.00052), amygdala (*p* = 0.0416), and cerebellum (*p* = 0.0006) (Table S1). Similarly, there was significantly elevated average expression within the deep gray nuclei (i.e., thalamus, caudate, putamen, pallidum) compared to hippocampus (*p* = 0.037) and cerebellum (*p* = 0.047). Average expression between deep gray nuclei and neocortical regions did not differ (*p* = 0.252).

### *PRNP* expression levels better correlate with imaging markers in early compared to late stage disease

**Figure 3:**
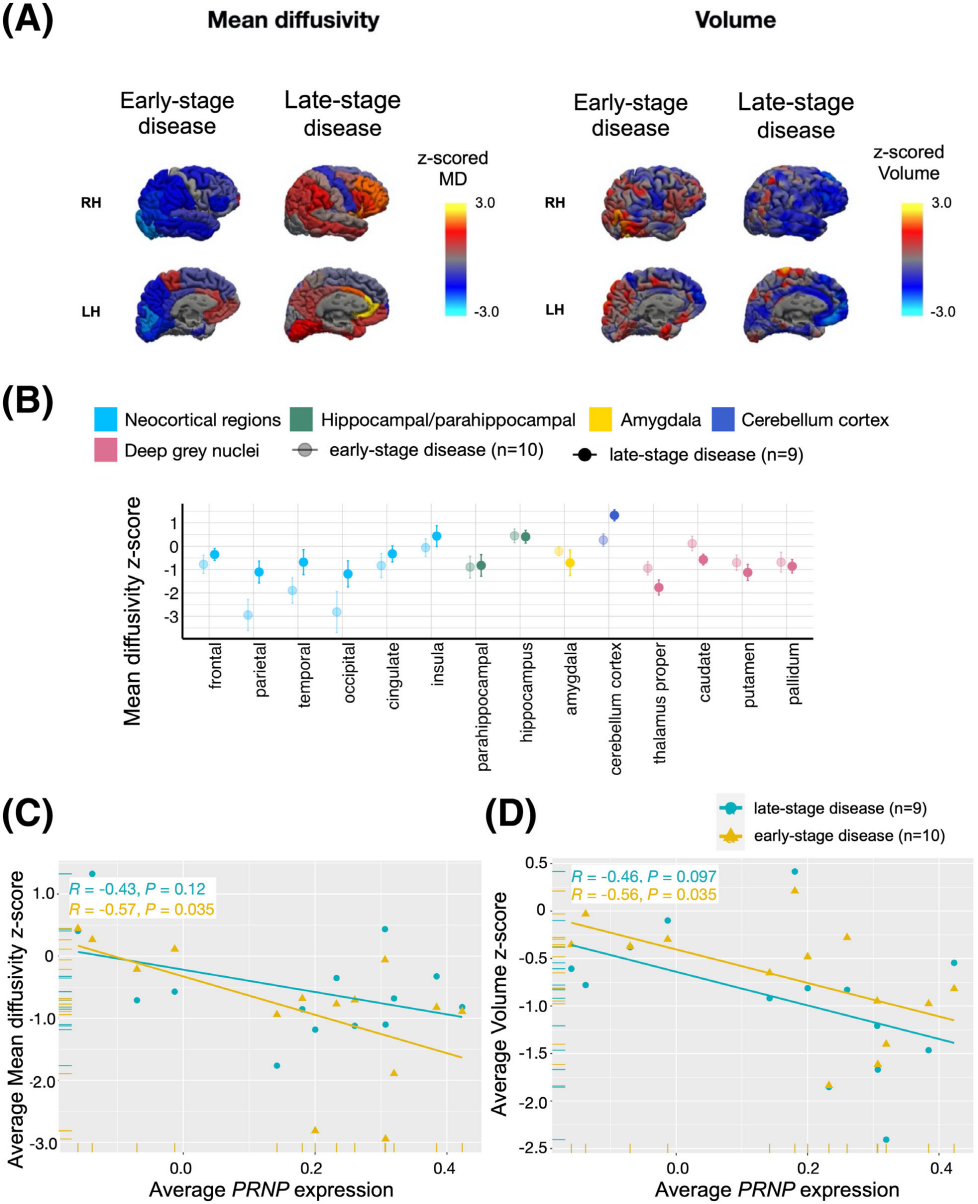
Change in mean diffusivity with disease progression. (A) Mean diffusivity and volume values for Desikan–Killiany atlas cortical ROIs mapped onto the cortical surface for two representative CJD patients, one with early‐stage disease (Barthel score of 100) and the other with late‐stage disease (Barthel score of 50). Blue hues show reduced mean‐diffusivity/volume and red‐yellow hues show increased mean‐diffusivity/volume. Mean diffusivity in involved areas is reduced in early‐stage disease but then increases or pseud‐normalizes in later stage disease when involved areas also show prominent volume loss. (B) Jitter boxplots showing absolute values of *z*‐scored cortical and subcortical regional mean diffusivity in patients with sCJD and healthy controls. Imaging measures are *z*‐scored within each region using the mean and standard deviation of healthy controls. The *x*‐axis is labeled with respect to brain structure. (C) Pearson correlations between averaged MD *z*‐score values and averaged *PRNP* expression across 14 brain regions in early‐stage disease and late‐stage sCJD disease. Disease stage was defined by Barthel scores. The linear relationships were non‐uniform with a strong negative relationship between average regional MD and *PRNP* expression in early‐stage disease and a much weaker relationship at later disease stage. (D) Pearson correlations between averaged volume and averaged *PRNP* expression across 14 brain regions in early‐stage disease and late‐stage sCJD disease. Disease stage was defined by Barthel scores. The relationship between volume and expression did not meet significance in the late‐stage group, although the slope of the relationship was similar, such that reduced volume was associated with higher PRNP expression in the same region in both early and late disease stage. CDR, clinical dementia rating; RH, right hemisphere; LH, left hemisphere; MD, mean diffusivity.

Figures 2B,C show how volume and mean diffusivity vary across cortical and subcortical regions in sCJD cases compared to healthy controls. On average, compared to controls, patients with sCJD showed reduced volume in the neocortex, parahippocampal gyrus, cerebellum, thalamus, and putamen (Fig. 2B; Table S2, *p* < 0.004) but did not differ in hippocampus, amygdala, caudate, or pallidum. Compared to controls, patients with sCJD showed decreased mean diffusivity in neocortical regions, parahippocampal gyrus, amygdala, thalamus, caudate, putamen, and pallidum (*p* < 0.007), increased mean diffusivity within cerebellum (*p* = 0.005), but no differences in mean diffusivity within the insula or hippocampus (Fig. 2C, Table S3).The boxplots in Fig. 2 show general correspondence between brain regions showing higher average *PRNP* expression and those showing greater average changes in regional volume and MD in patients with sCJD compared to controls. For example, both *PRNP* expression and sCJD associated imaging differences are strongest throughout the cerebral cortex and weakest in the hippocampus. However, many regions—particularly those with low average *PRNP* expression—showed more heterogeneous associations. In the case of MD, some of this heterogeneity likely relates to a phenomenon we have previously described whereby MD values in regions affected by sCJD change non‐linearly over time, such that MD in involved areas is initially reduced but then increases or pseudo‐normalizes as the disease progresses in that area.^14^, ^15^ In Fig. 3A we illustrate this concept by showing vertex wise changes in cortical volume and MD for representative sCJD patients from our cohort at early versus late stages of disease (defined by Barthel scores with early stage = 100, mid stage <100 and >55, and late stage ≤55^31^). For the patient with early‐stage disease, who had a Barthel score of 100, there is *reduced* MD throughout the cortex but also minimal cortical atrophy. Whereas, for the patient with late‐stage disease, who had a Barthel score of 55, there is *increased* MD across most of the cortex and marked cortical atrophy, particularly in frontal regions. Given that regions affected in sCJD can show either reduced or increased MD compared to controls depending on the stage of the disease in that region, and that our cohort is heterogeneous with respect to disease stage (Barthel scores ranged from 0 to 100), we separately evaluated regional MD and its relationship to regional *PRNP* expression for patients in the early and late stages of disease.

We first divided our cohort into equal‐sized tertiles, according to Barthel scores (early stage = 100, mid stage <100 and >55, late stage ≤55).^31^ To increase our power to detect differences between groups, we restricted our comparisons to patients in the early (*n* = 10) and late‐stages (*n* = 9) of disease. Regional patterns of MD in the early and late stages of disease differed from controls (Fig. 3B, values are below zero). sCJD cases at early stage of disease showed reduced MD compared to controls in neocortical regions, including frontal (*p* = 0.03), parietal (*p* = 0.0001), temporal (*p* = 0.001), and occipital (*p* = 0.02) lobes, and in deep gray nuclei, including thalamus (*p* = 0.0001) and putamen (*p* = 0.01). In comparison, sCJD cases in the late stages of disease showed no difference in MD in neocortical regions compared to controls, increased MD in insula (*p* = 0.02) and cerebellum cortex (*p* = 0.001), and decreased MD in deep gray nuclei—thalamus proper (*p* = 0.000006), caudate (*p* = 0.003), putamen (*p* = 0.0003), and pallidum (*p* = 0.0007). Further, regional patterns of MD in the early stages of disease differed from regional patterns of MD in late stage of disease, such that the late‐stage group showed increased MD in parietal (*p* = 0.03) and cerebellum (*p* = 0.005), and reduced MD in thalamus (*p* = 0.03).

We then correlated average *PRNP* expression with regional MD and volume separately for the early and late‐stage groups to assess whether imaging finding were correlated more with expression levels at a particular stage in the disease course. We found that regional *PRNP* expression significantly correlated with average MD and volume in early stage of disease (MD: *r* = −0.57, *p* = 0.035; volume: *r* = −0.56, *p* = 0.035) but not at the late stage of disease (MD: *r* = −0.43, *p* = 0.12; volume: *r* = −0.46, *p* = 0.097) (Fig. 3C,D). For volume, even though the relationship with expression did not meet significance in the late‐stage group, the slope of the relationship was similar, such that reduced volume was associated with higher *PRNP* expression in the same region in both early and late disease stage (see similar negative slopes of lines in Fig. 3D). For MD, the linear relationships were non‐uniform with a strong negative relationship between average regional MD and *PRNP* expression in early‐stage disease and a much weaker relationship at later disease stage (see flattening of slope for the relationship in Fig. 3C for late‐stage disease). This is in keeping with the concept that affected brain regions in sCJD show dynamic diffusion changes over the disease course and suggests that at the whole brain level reduced MD—the key imaging finding in early‐stage disease—is also the imaging feature most strongly associated with regional *PRNP* expression.

### Regional patterns of *PRNP* expression mirror imaging findings in sCJD at the individual level

**Figure 4:**
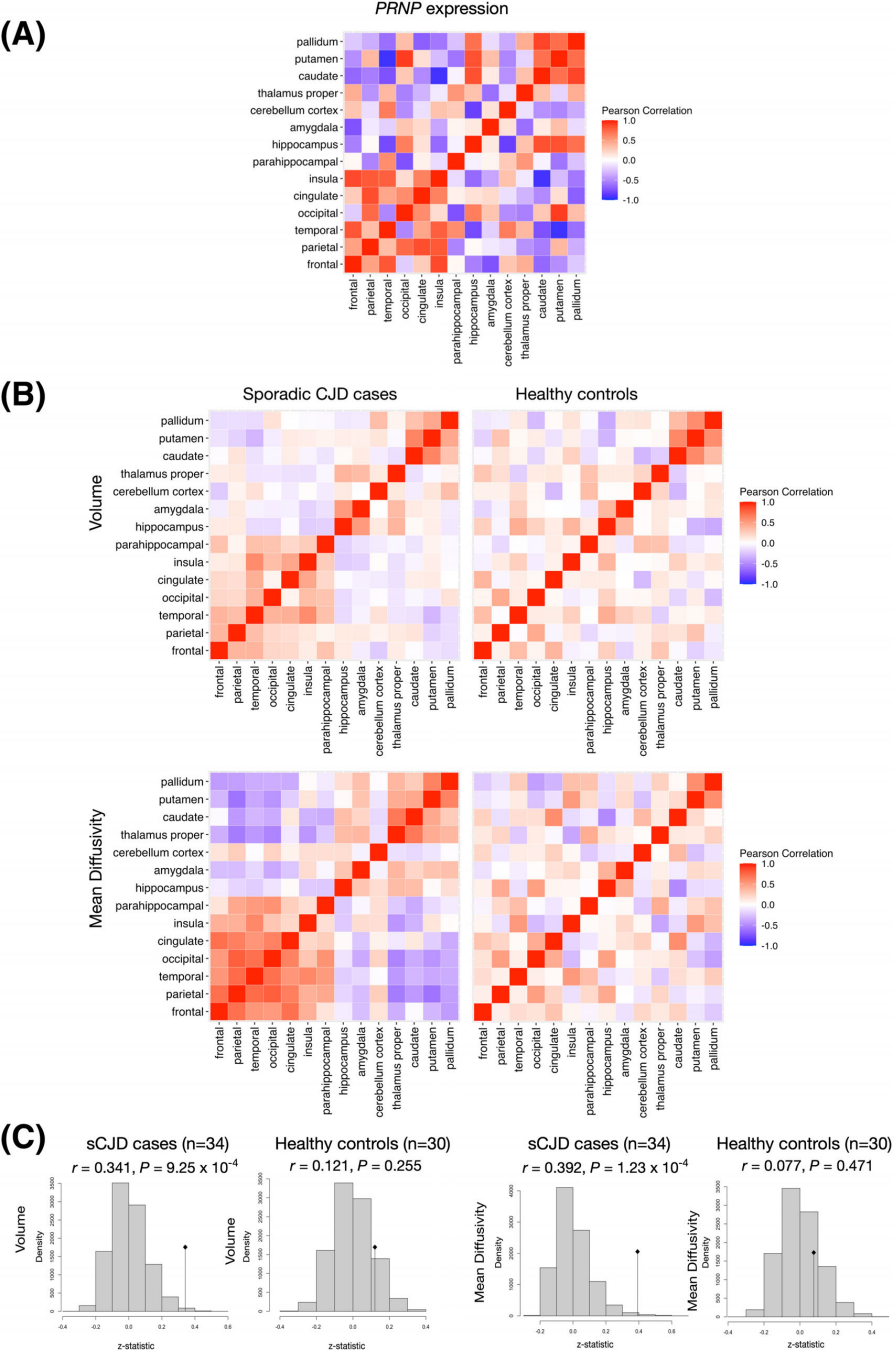
Partial correlation analysis shows relationships between regional patterns of *PRNP* gene expression and imaging measures. Partial correlation matrices of (A) *PRNP* expression in healthy individuals, and (B) Volume and mean diffusivity measures in sCJD cases (left) and healthy controls (right). (C) Mantel test evaluating similarity between regional patterns of correlation in *PRNP* gene expression and regional patterns of correlation in volume (top) and mean diffusivity (bottom) in CJD cases (left) compared to healthy controls (right). In each case the vertical line represents the observed *z*‐statistic compared to the distribution of values expected under the null hypothesis of chance similarity between the pairs of matrices, one‐tailed p‐values reflect the chance probability of observing a test statistic as extreme as that observed. Regional correlations in *PRNP* expression are similar to regional correlations in brain volume (*r* = 0.341, *p* = 9.25 × 10^−4^) and mean diffusivity (*r* = 0.392, *p* = 1.23 × 10^−4^) in sCJD cases but not in controls (volume: *r* = 0.121, *p* = 0.255; diffusivity *r* = 0.07, *p* = 0.471).

One of the goals of this manuscript is to highlight how group level analyses can obscure regional associations between imaging findings and *PRNP* expression levels. As demonstrated in the prior section, this can result from averaging across patients at different disease stages. However, any group level analysis will tend to obscure how expression and imaging markers co‐vary across regions within individual participants. Therefore, in our subsequent analyses, we directly measure the strength of regional correlations at the individual participant level, thus avoiding the dilution of these relationships that can occur at the group level. To test this, we explored regional *PRNP* expression across cortical and subcortical regions using partial correlation network analysis. The partial correlation matrix of regional *PRNP* expression in healthy brains revealed two positively correlated clusters of regions comprising most neocortical areas in one cluster and components of the basal ganglia in another (Fig. 4A). Individuals with higher *PRNP* expression within the frontal lobe also tended to have higher expression within the temporal, parietal, cingulate, and insular regions. Similarly, individuals with higher *PRNP* expression within the caudate had higher expression in the putamen and pallidum. Interestingly, *PRNP* expression within the cortical cluster was largely negatively correlated with expression in the basal ganglia. The exception to this pattern was the occipital cortex and hippocampus, where expression levels tended to be positively correlated with the basal ganglia and negatively correlated with other neocortical regions. Collectively, the findings in Figs. 2 and 4A highlight a pattern of *PRNP* expression characterized by lowest *PRNP* expression in the hippocampus, amygdala, and cerebellar cortex, positively correlated *PRNP* expression within the basal ganglia, positively correlated *PRNP* expression in most neocortical regions, and negatively correlated patterns of expression between the basal ganglia and most neocortical regions, except for occipital cortex.

We next asked whether regional patterns of *PRNP* expression in healthy individuals correlate with regional patterns in brain volume and mean diffusivity in patients with sCJD (Fig. 4B,C). Across the same brain regions, partial correlation analysis revealed that regional correlations in *PRNP* expression are similar to regional correlations in brain volume (*r* = 0.341, *p* = 9.25 × 10^−4^) and mean diffusivity (*r* = 0.392, *p* = 1.23 × 10^−4^) in sCJD cases and not similar to regional correlations in brain volume (*r* = 0.121, *p* = 0.255) or mean diffusivity (*r* = 0.07, *p* = 0.471) in healthy controls.

### Cortical patterns of *PRNP* expression correlate most strongly with *l*GI in sCJD

Our prior analysis suggests that regional patterns of *PRNP* expression in healthy individuals correlate with regional patterns of mean diffusivity and brain volume loss seen on imaging in sCJD patients, at least at the lobar level. For the cerebral cortex, the Allen Brain Atlas provides more finely sampled *PRNP* expression data, allowing us to test whether these associations hold at a higher spatial resolution. Focusing on the cortex, we can also ask whether more sophisticated measures of cortical morphology, such as local gyrification index (*l*GI)—which has been proposed as a more sensitive measure of morphological change in neurodegenerative diseases^28^, ^32^—better capture these associations than standard measures of cortical volume or thickness.

**Figure 5:**
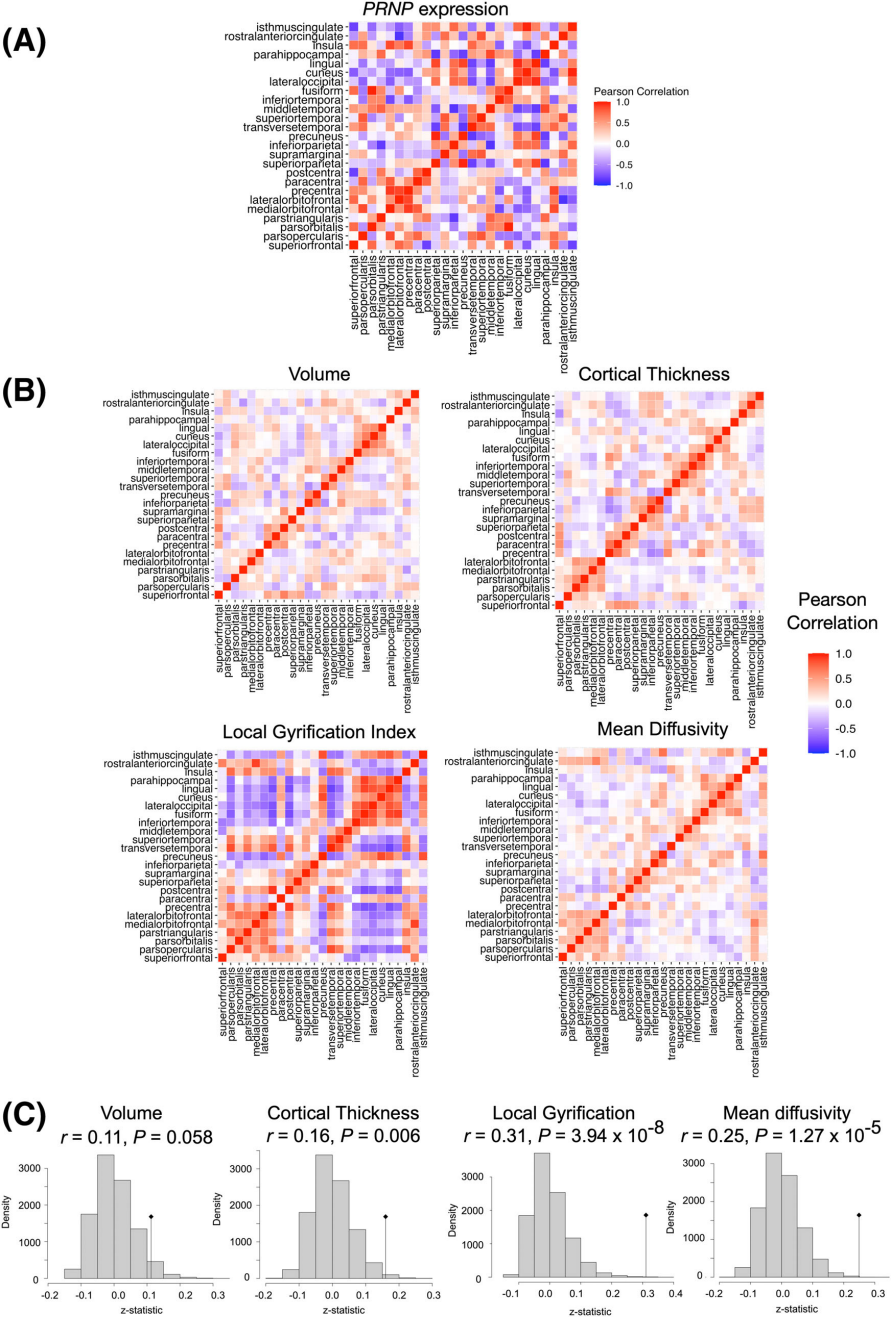
Partial correlation analysis shows relationships between regional patterns of *PRNP* gene expression and various imaging measures for a finer grained parcellation of the cerebral cortex. (A) Correlation matrix for regional PRNP expression in healthy controls (B) Correlation matrices for regional cortical volume, cortical thickness, local gyrification index, and mean diffusivity in sCJD cases. (C) The corresponding Mantel test evaluating similarity between the regional pattern of correlation for each imaging measure in sCJD cases and the regional pattern of correlation in *PRNP* gene expression. In each case, the vertical line represents observed *z*‐statistic compared to the distribution of values expected under the null hypothesis of chance similarity between the pairs of matrices, one‐tailed p‐values reflect the chance probability of observing a test statistic as extreme as that observed. As shown, for *l*GI and MD, the observed value of the Mantel test statistic (dark line) is far from the expected null distribution and highly significant (*p* = 3.94 × 10^−8^ and *p* = 4.57 × 10^−5^, respectively), showing strong correlation with the *PRNP* expression matrix.

Using this finer‐grained parcellation of cortical regions, we again found that regional correlations in imaging measures in patients with sCJD recapitulate regional correlations in *PRNP* expression (Fig. 5). For example, the correlation matrix for *PRNP* expression shows strong positive correlation within, and negative correlation between, clusters of frontal and occipital regions, including the medial and lateral orbitofrontal gyri in the frontal lobe, and lingula and cuneus in the occipital lobe (Fig. 5A). This same pattern is present in each of the imaging measures in patients with sCJD (Fig. 5B), but is strongest for *l*GI, followed by MD, and weakest for cortical thickness and volume (Fig. 5C).

**Figure 6:**
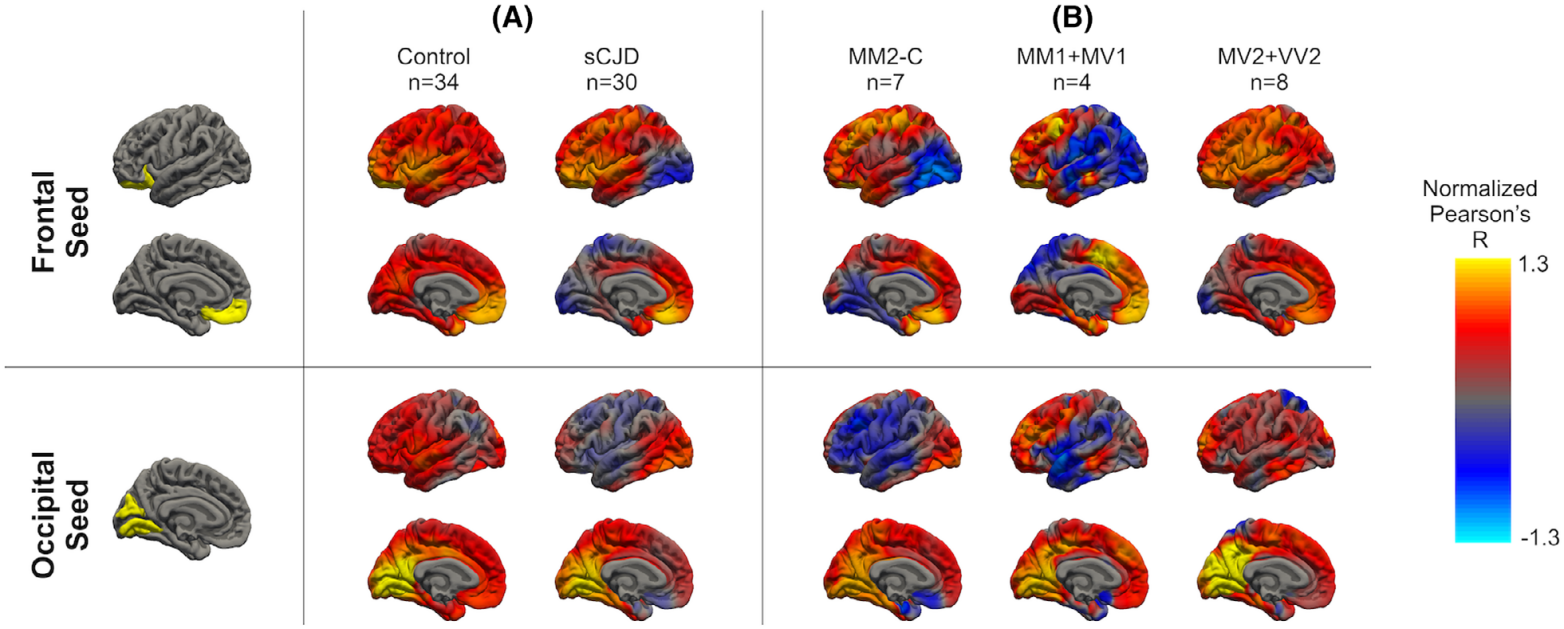
Average whole‐brain vertex‐wise correlations in local gyrification index using average *l*GI from the yellow‐shaded regions as a seed—frontal seed (top) and occipital seed (bottom). (A) sCJD cases (*n* = 34 cases) compared to healthy controls (*n* = 30 cases). (B) MM2‐C subtype of sCJD (*n* = 7 cases), compared to MM1/MV1 subtypes of sCJD (*n* = 4 cases), and MV2/VV2 subtypes of sCJD (*n* = 8 cases).

To further explore how *PRNP* expression‐related patterns of cortical anatomic correlation differ between sCJD cases and controls we conducted a post‐hoc surface‐based vertex‐wise analysis examining the correlation between average *l*GI within “seed” regions defined by the *PRNP* expression correlation matrix and *l*GI at every other vertex across the cerebral cortex.^28^ In these analyses, we focused on the strong positive correlation within, and negative correlation between, clusters of frontal and occipital regions seen in the *PRNP* expression correlation matrix (Fig. 5A), using average *l*GI within the medial and lateral orbitofrontal regions as the seed for the frontal cluster and average *l*GI within lingula and cuneus regions as the seed for the occipital cluster. These seed regions were chosen (somewhat arbitrarily) as the two most strongly correlated spatially contiguous regions within each of the corresponding clusters in the *PRNP* expression correlation matrix. This vertex‐wise analysis confirms the strong negative correlation between *l*GI in frontal and occipital regions in sCJD cases when compared to controls (Fig. 6A), recapitulating the pattern of *PRNP* expression seen in healthy brains (Fig. 5A).

Different molecular subtypes of sCJD differ in the degree to which cerebral cortex is affected both pathologically^33^, ^34^ and radiographically.^35^ For example, of the subtypes represented in our clinical population, MV2 and VV2 are known to primarily affect the deep gray nuclei and largely spare the cortex, in contrast, MM2‐C primarily affects the cortex, and MM1 and MV1 involve both cortical and subcortical structures. To investigate whether our *PRNP*‐expression related patterns of cortical anatomic correlation support this distinction, we repeated our surface‐based vertex‐wise analyses of *l*GI, separately for the 24 sCJD patients with molecular subtype group information, excluding 5 patients with mixed molecular subtypes. We found that the groups indeed exhibit distinct patterns of vertex‐wise correlation in *l*GI for the frontal and occipital seeds.

For the frontal seed (Fig. 6B, top), the MM2‐C and MM1/MV1 groups, for which imaging and pathology suggest prominent cortical (and for MM1/MV1 some subcortical) involvement, showed a strikingly different pattern to controls, with largely negative correlations between the frontal seed and posterior neocortical regions including the occipital cortex. In contrast, across much of the cortex the MV2/VV2 group, for which imaging and pathology suggest primarily subcortical involvement, showed a very similar pattern of positive correlation to controls, except for the occipital and inferior temporal lobes where correlations were weakly negative. For the occipital seed (Fig. 6B, bottom), we also found that correlation patterns differed across the MM2‐C, MM1/MV1, and MV2/VV2 groups. The MM2‐C and MM1/MV1 groups again showed pronounced differences compared to controls with heterogenous correlations between *l*GI of the occipital seed and anterior neocortical regions. For example, there were strong negative correlations with the subgenual cingulate, frontal operculum, insula, and perisylvian temporal and parietal lobes, which were stronger for the MM2‐C than the MM1/MV1 group, and positive correlations with other parts of frontal and posterior temporal cortex. The MV2/VV2 group, however, looked similar to controls with the exception of somewhat weaker positive correlations with frontal and temporal regions and negative correlations with superior parietal regions.

## Discussion

As an initial step toward developing quantitative imaging‐genetic correlations for sCJD, here we explored the relationship between patterns of regional brain expression of *PRNP*, the gene that encodes the cellular form of the prion protein (PrP^C^), and imaging findings in sCJD. We find that regional patterns of *PRNP* expression in the healthy brain correlate with regional patterns of classical imaging findings (including diffusion signal abnormalities and volume loss) in sCJD. Moreover, in the cerebral cortex of patients with sCJD, regional correlations in *l*GI—a higher order measure of cortical morphology—more closely matched regional correlations in *PRNP* expression than classical measures of cortical volume or thickness and this relationship was strongest for sCJD subtypes known to preferentially involve the cortex. Collectively, these findings raise the possibility that the genetic and molecular environment in which the prion protein is expressed creates variable vulnerability to misfolding across the brain and results in patterns of imaging findings that may provide markers for different disease subtypes.

To our knowledge, this is the first study to show a relationship between human *PRNP* expression and imaging findings in sCJD. In healthy controls, we found that average levels of *PRNP* expression are highest in brain regions commonly involved in sCJD, such as the cortex and putamen, and lowest in regions such as the hippocampus and amygdala, that are generally spared. In our sample of patients with sCJD compared to controls, average mean diffusivity and volume were decreased in brain regions with higher *PRNP* expression, but this association was strongest in patients with early compared to late stage disease. In the case of diffusion this effect of disease stage is likely due to the non‐linear changes in diffusion that occur within affected brain regions in sCJD over the disease course—starting with reduced diffusivity but later progressing to normal (or even increased) mean diffusivity.^15^, ^31^ Interestingly, a recent study that explored the relationship between regional patterns of diffusion in sCJD and normative measures of network structural connectivity reported no correlation between average regional diffusion measures in sCJD and average regional *PRNP* expression.^36^ That study relied on qualitative assessments of diffusion signal abnormality (presence or absence of reduced diffusivity, assessed as abnormal *hyperintense* signal, on clinical diffusion images) rather than quantitative measures. It seems likely that this apparent discrepancy between our results is due to a failure to consider this non‐linear diffusion effect—that abnormal diffusion in sCJD may manifest as reduced or increased mean diffusivity depending on the disease stage in an affected region, even within the same individual.

We further found that in healthy controls, *PRNP* expression levels show distinct regional patterns of correlation across the brain, with strong positive correlation among many cortical regions and among subcortical nuclei, but overall negative correlation between cortical and subcortical regions. In sCJD cases (but not controls), we found that regional correlations in imaging measures matched these regional correlations in *PRNP* expression. In the cortex, where *PRNP* expression data are available at higher spatial resolution, regional correlations in mean diffusivity and *l*GI showed the greatest similarity to *PRNP* expression including particularly strong correlations within and between two clusters of regions within the frontal and occipital lobes. Using these clusters as seeds in a post‐hoc whole brain vertex‐wise analysis, we examined how correlations in *l*GI vary continuously across the cortex in patients with sCJD compared to controls and between groups of patients with different molecular subtypes of sCJD. As expected from our regional analyses, these seeds produced very different cortical correlation maps in sCJD cases compared to controls. More interestingly, correlation maps also differed between different molecular subtypes of sCJD. Subtypes known to predominantly involve the cortex (MM2‐cortical) differed most from controls, followed by subtypes known to have mixed cortical and subcortical (MM1/MV1) or mostly subcortical (MV2/VV2) involvement, the last looking very similar to controls. These vertex‐wise results suggest that subtypes known to have greater cortical involvement show patterns of regional anatomic (*l*GI) correlation across the cerebral cortex that more closely mirror regional correlations in *PRNP* expression, supporting the idea that different molecular subtypes of sCJD have both distinct clinical phenotypic characteristics^5^, ^6^ and distinct imaging profiles.^16^, ^30^

Our results suggest that among measures of cortical morphology cortical gyrification (*l*GI) better captures neuroanatomic differences in sCJD compared to more traditional measures of cortical thickness and volume. Cortical gyrification is the process that creates the folded shape of the human brain with distinct sulci and gyri, allowing for increase cortical surface area and neuronal density within a limited intracranial volume. A complex cortical folding pattern also allows functionally similar regions to be closer together, reducing the length of interconnecting fiber tracts and facilitating efficient cortico‐cortical communication.^37^, ^38^ Greater cortical gyrification in healthy mid‐life adults has been associated with better cognitive performance^37^ and reduced cortical gyrification has been found in regions affected in Parkinson's^39^ and Alzheimer's disease.^40^ Similarly, presymptomatic individuals with hexanucleotide repeat expansion in *C9ORF72*—the most common genetic cause of familial and sporadic frontotemporal dementia (FTD) and amyotrophic lateral sclerosis (ALS)—show reduced *l*GI in regions known to be affected in ALS/FTD decades before symptom onset.^32^ In each case these findings were specific to *l*GI compared to cortical thickness or volume, and collectively these results suggest *l*GI is a useful marker for detecting regional patterns of brain atrophy in patients with sCJD and other neurodegenerative diseases.

The precise function(s) of PrP^C^, the membrane‐associated protein product of *PRNP* which is found in neurons, astrocytes, and other mammalian tissues,^41^ is still unknown. Nevertheless, PrP^C^ is required for prion disease—mice lacking PrP^C^ are resistant to prion infection,^42^ and neurons lacking PrP^C^ do not develop pathologic changes even when chronically exposed to misfolded PrP^Sc^.^43^ Prions are thought to propagate in the brain through a process similar to crystal growth with an initial “seed” of misfolded PrP^Sc^ catalyzing the conversion of native PrP^C^ to the abnormal form.^2^ It has recently been suggested that such propagation in neurodegenerative proteinopathies may involve trans‐neuronal spread of misfolded protein between interconnected brain regions and may therefore be spatially constrained by structural connectivity across the brain.^36^, ^44^ Whether because of increased susceptibility to PrP^C^ misfolding, increased PrP^Sc^ propagation, or increased vulnerability to PrP^Sc^ related neuronal damage, our findings suggest that brain regions that show correlated levels of *PRNP* expression also define networks that have selective vulnerability to CJD pathology.^45^

Our overall findings point to the importance of regional expression of *PRNP* in forming the genetic landscape on which the pathobiology of sCJD unfolds and raises the possibility that multiple genetic or environmental factors may be important in determining the regional characteristics of different sCJD subtypes. Currently, the specifics of these other factors are unknown. Our results, however, raise the possibility that the distinct imaging patterns associated with different clinicopathologic subtypes of sCJD may reflect the interaction between regional patterns of *PRNP* expression and those of other gene networks, such as neuroinflammation‐associated genes, that may influence CJD pathobiology.^46^ This, in turn, suggests that screening for genes with a similar pattern of regional expression to *PRNP* may be one way of identifying other key members of this gene network and may provide insights into distinct mechanisms underlying different molecular subtypes of sCJD.

Our study has several limitations. First, our expression data come from healthy individuals, not patients with sCJD. It is important to define the normal underlying genetic landscape in which diseases like sCJD unfold, however, future work will need to investigate whether regional patterns of *PRNP* expression in sCJD cases are the same as those in healthy individuals. In addition, we cannot assume a one‐to‐one correspondence between gene expression levels and resultant protein levels in patients with sCJD, for example, regional PrP^C^ levels have been shown to differ between controls and patients with different neurodegenerative conditions.^47^ Second, it is well‐known that *PRNP* genotype (codon 129 polymorphism) and the molecular subtype of the native prion protein (PrP^C^) affect multiple aspects of prion disease, including clinical presentation, incubation period, and pathological phenotype;^48^, ^49^ however, the codon 129 genotype and molecular subtype of the six healthy participants used for the microarray expression analysis are unknown. Ultimately, simultaneous examination of *PRNP* expression, PrP^C^ levels/molecular subtypes, and imaging findings in patients with sCJD will be needed to understand how differential *PRNP* expression relates to changes in PrP^C^ levels and imaging findings in individual patients. Third, our molecular subtype analysis is limited by the small number of individuals from each subtype in our cohort of 34 sCJD cases (and did not include VV1 cases). Finally, although all molecular subtypes (except VV1) are represented in our cohort, our cohort has different proportions of the three codon 129 subtypes than large multinational cohorts, including fewer of the faster progressing subtypes (MM1 and VV2) which are typically the most prevalent sCJD group.^50^ Correspondingly, our cohort had a median Barthel score of 70/100, compatible with mild to moderate functional impairment. For these reasons, the imaging characteristics of our cohort may not be fully representative of the broader group of patients with sCJD and our results might be more relevant to the slower progressing group of patients or those in less advanced stages of disease, regardless of molecular subtype.

In conclusion, we found that regional patterns of *PRNP* expression in the healthy brain correlate with regional patterns of classical imaging findings in sCJD. Building on prior work, we found that different genetic‐molecular subtypes of sCJD are associated with distinct regional patterns of imaging findings and that cortical *l*GI might provide a more reliable imaging metric than cortical volume or thickness for quantifying morphological patterns of disease relevant to different subtypes of sCJD. Definitive diagnosis of CJD requires pathologic analysis of brain tissue.^51^ New amplification techniques, such as Real time quake‐induced conversion assay (RT‐QuIC), may enable premorbid diagnosis with high specificity through the detection of trace amounts of PrP^Sc^ in CSF and other bio‐samples;^52^ however, in current practice, MRI—and in particular diffusion weighted imaging—still forms a key part of the diagnostic criteria for probable sCJD. Our findings have implications for developing novel imaging markers to improve diagnostic classification between sCJD and other dementias and among different sCJD subtypes. Moreover, improved understanding of the genetic environment in which misfolding of the prion protein occurs, and how it might render some brain regions more vulnerable to sCJD, has the potential to improve our understanding of the underlying pathophysiology of sCJD.

## Author Contributions

IJB, LPS, and MG contributed to the concept and design of the study, analysis, and drafting a significant portion of the manuscript and figures. RSD contributed to the concept and design of the study. RMN contributed to analysis of the data and generation of figures, SS contributed to the analysis, and EC and MP contributed to acquisition and analysis of data.

## Conflicts of Interest

The authors report no competing interests related to this paper. Dr. Geschwind receives or received research support on prion disease from the NIH/NIA (grant R01 AG AG031189; R01AG062562; R56 AG055619) and the Michael J. Homer Family Fund. He has consulted for Adept Field Consulting (Backay Consulting), Ascel Health LLC, Anderson Boutwell Traylor, Best Doctors Inc., Biohaven Pharma Inc., Bioscience Pharma Partners, LLC (BPP), Clarion Consulting, First Thought Consulting, Grand Rounds Inc./UCSF Second Opinion Inc., Maupin Cox Legoy, Quest Diagnostics, Smith & Hennessey LLC and Trinity Partners LLC. He has received speaking honoraria for various medical center lectures, Oakstone Publishing and Wolters Kluwer. He has received past research support from Alliance Biosecure, CurePSP, the Tau Consortium, Quest Diagnostics, and NIH. Dr. Geschwind serves on the board of directors for San Francisco Bay Area Physicians for Social Responsibility and on the editorial board of Dementia & Neuropsychologia.

## Supporting information


Table S1:
