# Supplementary material for: PRNP expression predicts imaging findings in sporadic Creutzfeldt‐Jakob disease
Source: Ann Clin Transl Neurol. 2023 Feb 6;10(4):536–52. doi: 10.1002/acn3.51739 (PMC10109249; doi:10.1002/acn3.51739)
Supplement: Supplementary file 1 — Table S1: [file ACN3-10-536-s001.docx]

Supplementary Table 1

| **Average PRNP expression neocortical regions compared to all other regions** | | | | | |
| --- | --- | --- | --- | --- | --- |
| Brain Region | Mean Neocortex | Mean Region | t -statistic | *P* | *P* adjusted |
| subcortex | 0.292 | 0.142 | 1.232 | 2.52E-01 | 2.52E-01 |
| hippocampus | 0.292 | -0.160 | 5.028 | **5.16E-04** | **1.51E-03** |
| parahippocampal | 0.292 | 0.423 | -1.291 | 2.27E-01 | 2.52E-01 |
| amygdala | 0.292 | -0.070 | 2.479 | **4.16E-02** | 6.93E-02 |
| cerebellum.cortex | 0.292 | -0.138 | 4.923 | **6.04E-04** | **1.51E-03** |
|  |  |  |  |  |  |
|  |  |  |  |  |  |
| **Average *PRNP* expression in subcortical regions compared to all other regions** | | | | | |
| Brain Region | Mean Neocortex | Mean Region | t -statistic | *P* | *P* adjusted |
| neocortex | 0.142 | 0.292 | -1.232 | 2.52E-01 | 2.52E-01 |
| hippocampus | 0.142 | -0.160 | 2.481 | **3.68E-02** | 9.91E-02 |
| parahippocampal | 0.142 | 0.423 | -2.143 | 5.94E-02 | 9.91E-02 |
| amygdala | 0.142 | -0.070 | 1.269 | 2.35E-01 | 2.52E-01 |
| cerebellum.cortex | 0.142 | -0.138 | 2.338 | **4.73E-02** | 9.91E-02 |
|  |  |  |  |  |  |
| **Average *PRNP* expression in hippocampus compared to all other regions** | | | | | |
| Brain Region | Mean Neocortex | Mean Region | t -statistic | *P* | *P* adjusted |
| neocortex | -0.160 | 0.292 | -5.028 | **5.16E-04** | **1.29E-03** |
| subcortex | -0.160 | 0.142 | -2.481 | **3.68E-02** | 6.14E-02 |
| parahippocampal | -0.160 | 0.423 | -5.668 | **2.47E-04** | **1.24E-03** |
| amygdala | -0.160 | -0.070 | -0.610 | 5.60E-01 | 7.01E-01 |
| cerebellum.cortex | -0.160 | -0.138 | -0.246 | 8.11E-01 | 8.11E-01 |

Bold, *P* < .05

| Supplementary Table 2 | |  |  |  |  |
| --- | --- | --- | --- | --- | --- |
| **Average brain volume in sCJD cases compared to healthy controls** | | | | | |
|  | **beta** | **se** | ***t*** | ***P*** | ***P* adjusted** |
| frontal | -1.819 | 0.185 | -9.856 | **2.83E-17** | **3.97E-16** |
| parietal | -1.628 | 0.191 | -8.539 | **4.09E-14** | **2.86E-13** |
| temporal | -2.322 | 0.305 | -7.615 | **5.83E-12** | **2.72E-11** |
| occipital | -1.133 | 0.247 | -4.593 | **1.06E-05** | **2.12E-05** |
| cingulate | -1.334 | 0.206 | -6.480 | **1.95E-09** | **6.82E-09** |
| insula | -1.488 | 0.239 | -6.222 | **6.90E-09** | **1.93E-08** |
| parahippocampal | -0.849 | 0.243 | -3.492 | **6.66E-04** | **1.16E-03** |
| hippocampus | -0.287 | 0.171 | -1.678 | 9.60E-02 | 1.22E-01 |
| amygdala | -0.249 | 0.229 | -1.090 | 2.78E-01 | 2.78E-01 |
| cerebellum cortex | -0.584 | 0.190 | -3.083 | **2.53E-03** | **3.54E-03** |
| thalamus proper | -0.857 | 0.185 | -4.624 | **9.32E-06** | **2.12E-05** |
| caudate | -0.238 | 0.206 | -1.155 | 2.50E-01 | 2.69E-01 |
| putamen | -0.666 | 0.195 | -3.418 | **8.53E-04** | **1.33E-03** |
| pallidum | -0.295 | 0.240 | -1.228 | 2.22E-01 | 2.59E-01 |

Bold, *P* < .05

| Supplementary Table 3 | | | |  |  |  |
| --- | --- | --- | --- | --- | --- | --- |
| **Average mean diffusivity values in sCJD cases compared to healthy controls** | | | | | | |
|  | **beta** | **se** | ***t*** | | ***P*** | ***P* adjusted** |
| frontal | -0.827 | 0.246 | -3.357 | | **1.05E-03** | **1.63E-03** |
| parietal | -1.953 | 0.382 | -5.114 | | **1.16E-06** | **4.07E-06** |
| temporal | -1.664 | 0.353 | -4.714 | | **6.43E-06** | **1.80E-05** |
| occipital | -1.952 | 0.456 | -4.285 | | **3.63E-05** | **7.27E-05** |
| cingulate | -1.009 | 0.275 | -3.675 | | **3.52E-04** | **6.17E-04** |
| insula | -0.332 | 0.299 | -1.110 | | 2.69E-01 | 2.90E-01 |
| parahippocampal | -0.820 | 0.255 | -3.223 | | **1.62E-03** | **2.27E-03** |
| hippocampus | 0.142 | 0.196 | 0.727 | | 4.69E-01 | 4.69E-01 |
| amygdala | -0.711 | 0.241 | -2.946 | | **3.85E-03** | **4.90E-03** |
| cerebellum cortex | 0.573 | 0.203 | 2.831 | | **5.42E-03** | **6.32E-03** |
| thalamus proper | -2.020 | 0.275 | -7.346 | | **2.38E-11** | **3.33E-10** |
| caudate | -0.887 | 0.205 | -4.325 | | **3.10E-05** | **7.24E-05** |
| putamen | -1.893 | 0.280 | -6.760 | | **4.81E-10** | **3.37E-09** |
| pallidum | -1.178 | 0.224 | -5.260 | | **6.11E-07** | **2.85E-06** |

Bold, *P* < .05
